# Role of Diuretics and Ultrafiltration in Congestive Heart Failure

**DOI:** 10.3390/ph6070851

**Published:** 2013-07-04

**Authors:** Dmitry Shchekochikhin, Fawaz Al Ammary, JoAnn Lindenfeld, Robert Schrier

**Affiliations:** University of Colorado Division of Renal Diseases and Hypertension, 12700 East 19th Avenue, C281, Aurora, CO 80045, USA

**Keywords:** cardiac failure, diuretic resistance, diuretic combinations, ultrafiltration

## Abstract

Volume overload in heart failure (HF) results from neurohumoral activation causing renal sodium and water retention secondary to arterial underfilling. Volume overload not only causes signs and symptoms of congestion, but can impact myocardial remodeling and HF progression. Thus, treating congestion is a cornerstone of HF management. Loop diuretics are the most commonly used drugs in this setting. However, up to 30% of the patients with decompensated HF present with loop-diuretic resistance. A universally accepted definition of loop diuretic resistance, however, is lacking. Several approaches to treat diuretic-resistant HF are available, including addition of distal acting thiazide diuretics, natriuretic doses of mineralocorticoid receptor antagonists (MRAs), or vasoactive drugs. Slow continuous veno-venous ultrafiltration is another option. Ultrafiltration, if it is started early in the course of HF decompensation, may result in prominent decongestion and a reduction in re-hospitalization. On the other hand, ultrafiltration in HF patients with worsening renal function and volume overload after aggressive treatment with loop diuretics, failed to show benefit compared to a stepwise pharmacological approach, including diuretics and vasoactive drugs. Early detection of congested HF patients for ultrafiltration treatment might improve decongestion and reduce readmission. However, the best patient characteristics and best timing of ultrafiltration requires further evaluation in randomized controlled studies.

## 1. Introduction

Heart failure (HF) affects more that 5 million Americans and is the cause of nearly one million hospitalizations per year in the United States [1,2]. The main cause of HF hospitalization is symptomatic congestion. Prognosis after heart failure hospitalization is poor, with 50% of patients rehospitalized within 6 months and 25% to 35% mortality at 1 year [3]. Results from the Acute Heart Failure Registry (ADHERE) revealed that 33% of the patients were discharged with a weight loss of 5 pound or less, 16% were discharged with an increase in body weight and 30% were considered to be resistant to loop diuretics. Nearly 50% of patients still had symptoms of congestion at discharge [4].

**Figure 1:**
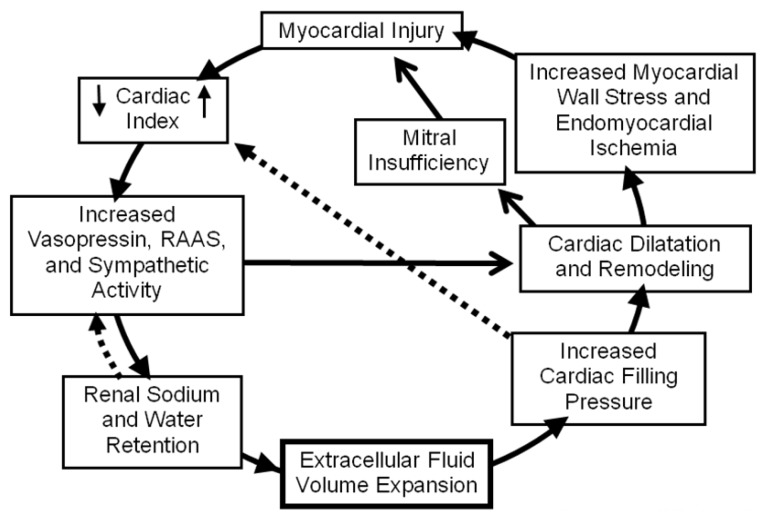
Vicious cycle of chronic heart failure. Reproduced from [8] with permission.

Sodium and water retention, the hallmark of HF, results in symptoms of pulmonary congestion (dyspnea, orthopnea, paroxysmal nocturnal dyspnea) and systemic venous congestion (edema, ascites, and hepatomegaly). Increased left ventricular filling pressures in the absence of clinical symptoms, so-called hemodynamic congestion, predicts subsequent clinical HF decompensation [5]. Studies using implantable intracardiac pressure sensors have demonstrated that left ventricular filling pressures are elevated for 3-4 weeks prior to a hospitalization for acute decompensated HF. Thus it is likely that chronically elevated ventricular filling pressures play a pivotal role in cardiac remodeling due to neurohormonal activation, increased myocardial wall stress, increased myocardial oxygen demands with ischemia, and increased mitral regurgitation [6,7].These events can result in a vicious cycle of cardiac output reduction with progressive renal salt and water retention (Figure 1) [8]. Reducing congestion, therefore, is a cornerstone of HF treatment. This review focuses on available approaches to treat congestion in HF patients.

## 2. Pathophysiology of Sodium and Water Retention in Heart Failure

**Figure 2:**
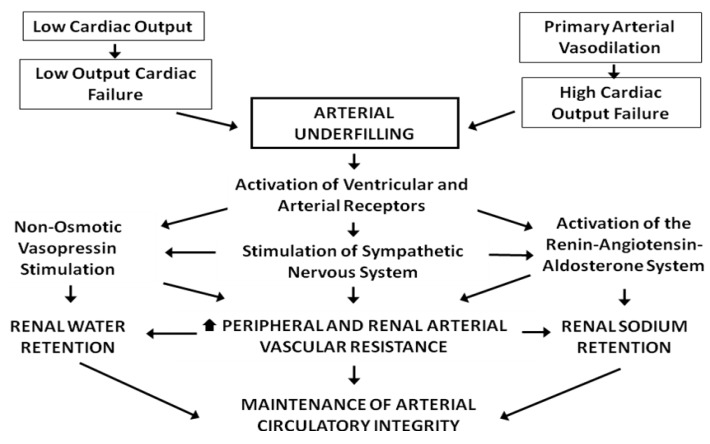
Pathogenesis of low and high cardiac output heart failure. Reproduced from [12] with permission.

In normal subjects, cross-talk between the heart and kidneys occurs through atrial-renal reflexes, which work to maintain total body volume in the normal range [8]. An increase in atrial pressure suppresses release of arginine vasopressin (AVP) through the Henry-Gauer Reflex and decreases renal sympathetic tone [9]. The increase in filling pressures in atria and ventricles also results in the release of natriuretic peptides (ANP and BNP) [10]. The result of these atrial-renal reflexes is to increase renal sodium and water excretion. However, in the setting of HF these normal responses are attenuated by decreased effective arterial volume, or so-called arterial underfilling [11]. Underfilling of the arterial circulation occurs because of a decrease in cardiac output in low-output HF and primary arterial vasodilatation in high-output HF. In both types of HF the inhibitory effects of the arterial stretch baroreceptors on the neurohumoral systems (renin-angiotensin-aldosterone system, catecholamines and AVP) are decreased. As shown in Figure 2, this results in vasoconstriction of systemic and intrarenal arterioles, increased sodium reabsorption and AVP mediated water retention [12].

**Figure 3:**
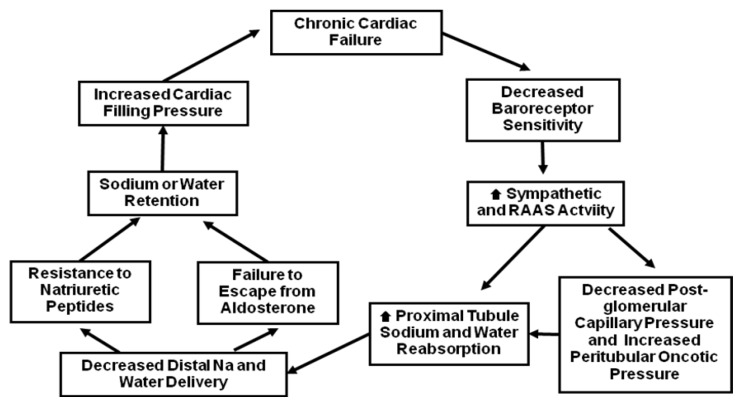
Vicious cycle of sodium and water retention in chronic heart failure. Reproduced from [8] with permission.

Angiotensin II (Ang II) causes increase in thirst, stimulation of the sympathetic nervous system, systemic and renal vasoconstriction, and stimulation of the synthesis of aldosterone [8,13,14,15]. Normally, the sodium retaining ability of aldosterone is temporary, and does not cause edema. This is because the increase in vascular volume, particularly in the arterial circulation, enhances sodium delivery to distal renal tubules which overrides the sodium retaining effect of aldosterone within approximately 3 days (“aldosterone escape”) [13,14]. In contrast, in patients with HF this “aldosterone escape” is impaired by a decrease in sodium delivery to the mineralocorticoid receptors in the distal nephron [13,14]. Intrarenal vasoconstriction with increased proximal sodium and water reabsorption also attenuates the salt losing action of natriuretic peptides in distal tubules [8,16] (Figure 3). Sympathetic stimulation also contributes to sodium and water retention by enhancing reabsorption and activating renin-angiotensin system (RAS) [8]. The vasoconstrictive effect of angiotensin II on the glomerular efferent arterioles decreases postglomerular capillary pressure and the resultant rise in peritubular oncotic pressure further enhances proximal tubular sodium reabsorption.

AVP, the antidiuretic hormone, is secreted from posterior pituitary gland in response to increased plasma osmolality or the non-osmotic effect of arterial underfilling [17]. Activation of vasopressin V1 receptors results in an increase in systemic vascular resistance. The non-osmotic stimulation of AVP activates the V2 receptor; this increases electrolyte-free water reabsorption in the renal collecting ducts.

In patients with decompensated HF an increase in renal venous pressure due to volume overload leads to increase in intrarenal vasoconstriction, activation of RAAS and enhanced proximal sodium and water reabsorption with further congestion. 

These different mechanisms of sodium and water retention in HF suggest a number of therapeutic options to reduce congestion. Dietary sodium restriction and use of loop diuretics that block sodium and water reabsorption in the proximal nephron are the cornerstones of therapy. Other strategies include diuretics that block sodium and water absorption in different segments of nephron. Increasing cardiac output by using intravenous vasodilators and/or inotropes may be effective if hypotension and reduced renal artery perfusion can be avoided. Extracorporeal ultrafiltration may also be effective. 

## 3. Use of Loop Diuretics to Treat HF

Loop diuretics (e.g., furosemide, torsemide, bumetanide and ethacrynic acid) are the main agents used to treat volume overload in HF. According to the ADHERE registry, 90% of patients with decompensated HF received intravenous loop diuretics [4]. The pharmacodynamics and pharmacokinetics of loop diuretics are presented in Table 1. These agents act by blocking the Na/K/2Cl cotransporter in the thick ascending loop of Henle. Loop diuretics are highly bound to serum proteins and to be active require secretion into the proximal tubule. This Na/K/2Cl pump is located on the luminal side of the nephron. Loop diuretics therefore must reach the tubular fluid to be active [18,19,20]. Therefore, in patients with severe renal insufficiency (e.g., GFR < 15 mL/min) larger doses of loop diuretics are required to achieve effective concentrations [21]. The various loop diuretics differ in intestinal absorption, especially with an edematous bowel wall present in decompensated HF. Bumetanide and torsemide are high absorbed (100% and 80% respectively) [21]. However, oral absorption of furosemide may vary from patient to patient as much as 10% to 100% [21,22]. Loop diuretics also differ in their half-lives, which determine the frequency of administration. Thus, once a day administration of an agent with a short half-life such as furosemide, could cause “rebound” sodium retention due to reabsorption of filtered sodium when there is no longer a diuretic agent present in the tubular lumen. This is especially true when a patient ingests sodium after the end of diuretic dosing interval [18,19,23]. Of note, once the plateau of maximal natriuretic response is achieved, further increase in dosing would fail to enhance the effect. Thus, in HF patients with preserved renal function large doses of diuretics may not be necessary. Thus, while a large single daily dose of a loop diuretic may not decrease congestion in HF, smaller doses given 2-3 times per day may be effective. 

**Table 1:** Pharmacology of loop diuretics with permission from Reference 18.

	Furosemide	Bumetamide	Torsemide
Relative IV patency (mg)	40	1	20
Bioavailability (%)	10-100 (50)	80-100	80-100
Average effect duration (h)	6–8	4–6	6–8
Oral to IV conversion	2:1	1:1	1:1
30 day cost ($)	4	4	19–23

The route of diuretic use in case of decompensated HF (bolus or continuous infusion) has been assessed in several clinical studies. A continuous infusion is designed to maintain stable amount of diuretic at the luminal site of action. Several small studies have suggested a benefit of continuous infusion of loop diuretics versus intermittent bolus doses. However, a recent Cochrane analysis suggested that currently available data were inadequate to support this contention [24].

The mode and dose of loop diuretics in decompensated HF have been evaluated in the randomized double-blind controlled trial Diuretic Optimization Strategies Evaluation (DOSE). The DOSE study demonstrated that there was no significant difference in global symptom relief or change in renal function at 72 h between intermittent versus continuous infusion of furosemide or between low dose (outpatient dose) versus high dose (2.5 times outpatient dose) of furosemide [25]. Later the weight loss was greater with the larger dose, however, with the 60-day follow-up there were no significant differences in outcomes between groups.

Nevertheless, continuous infusion of loop diuretics could be an option for HF patients who are unresponsive to initial bolus doses. The HF Society of America guideline for decompensated HF recommends switching from bolus to continuous infusion of diuretics in patients who appear to be nonresponsive to diuretics [26]. However, this approach needs to be assessed in randomized studies.

## 4. Use of Thiazide Diuretics to Treat HF

Thiazide diuretics act by inhibition of Na/Cl cotransporter in distal convoluted tubule. In general, these agents are weaker diuretics compared to loop-acting agents. Nevertheless, some patients with mild to moderate HF and preserved renal function can maintain fluid balance with thiazide diuretics. Thiazide diuretics are used in combination with loop diuretics when there is a poor natriuretic response to loop diuretics alone [27]. Chronic treatment with loop diuretics can result in renal adaptation, which includes hypertrophy and hyperfunction of distal tubular cells with enhanced sodium uptake in addition to the stimulation of aldosterone secretion [28,29]. Blocking distal tubule sodium reabsorption with thiazide diuretics can antagonize this renal adaptation to chronic loop diuretics [29,30]. There are several studies evaluating the combination of thiazide and loop diuretics. However, the total reported experience of this combination is limited to 300 HF patients [27]. Metolazone, a thiazide-like diuretic, is believed to be superior to other thiazides, due to additional inhibition of proximal tubule function [31]. A randomized double-blind study found no superiority of metolazone compared to bendroflumethiazide [32]. However, a response to metolazone plus furosemide was documented in a single patient resistant to chlorothiazide plus furosemide [33]. In addition to metolazone, improved natriuretic response to loop diuretics has been demonstrated using chlorothiazide, hydrochlorothiazide, quinethazone, indapamide, bendroflumethiazide, and butizide [27]. Moreover, thiazide diuretics are effective in enhancing the response to loop diuretics even in patients with advanced renal failure [27]. Metalozone, however, has a variable absorption and long half-life (about 2 days), which make other thiazides easier to use [18,21]. Theoretically, thiazide diuretics should be given at least 30 min before the loop diuretics in order to inhibit distal sodium reabsorption at the time the loop diuretics block proximal sodium reabsorption in the loop of Henle; however this strategy of diuretic administration has not been studied. In most studies reporting benefits of thiazide-loop diuretic combination, the 2 drugs were administrated at the same time [27]. Thiazide diuretics also act from the luminal side of the nephron. Thus, in cases of renal insufficiency larger doses are necessary to obtain effective urinary concentrations [18].

## 5. Use of Acetazolamide to Treat HF

Acetazolamide is a carbonic anhydrase inhibitor, acting primarily in the proximal tubule. Used alone, it is a weak diuretic due to compensatory distal reabsorption of sodium and water [18], however, acetazolamide produces an alkaline diuresis, thus normalizing hypochloremic alkalosis due to other diuretic use [34]. This approach is helpful in HF patients who should not receive saline to correct their metabolic alkalosis. With acetazolamide there must be caution with respect to worsening hypokalemia. The ability of acetazolamide to stimulate the respiratory system and reverse central sleep breathing abnormalities in HF patients has been demonstrated [35].

Acetazolamide can be an effective addition in loop diuretic resistant cases. Again, however, plasma potassium needs to be carefully monitored to avoid hypokalemia. Randomized trials of acetazolamide and loop diuretics in patients with decompensated HF would be important.

## 6. Use of mineralocorticoid receptor antagonists (MRAs) to treat HF

MRAs, spironolactone and eplerenone, have been shown to improve morbidity and mortality in HF patients [36,37,38]. However, the doses of MRAs used in these trials were low. In a dose ranging study prior to the Randomized ALdactone Evaluation Study (RALES) the investigators demonstrated that 25 mg/day of spironolactone did not decrease sodium retention [39]. The beneficial effect of 25 mg of spironolactone on HF survival in the RALES study was therefore due to blocking the non-genomic effects of aldosterone including cardiac inflammation, fibrosis and apoptosis [36]. Natriuretic doses of MRAs, *i.e.*, greater than 25 mg/day of spironolactone or 50 mg of eplerenone, are generally not used in HF patients, due to the risk of hyperkalemia. An association between hospitalization and hyperkalemia after publication of the RALES study was reported in a retrospective observational study from Canada [40]. However, a more recent large study from Scotland over the same time period did not find any increase in hospitalizations associated with a similar increase in prescribing MRAs [41]. 

The use of natriuretic doses of MRAs (e.g., spironolactone 50–100 mg) therefore could be a reasonable option to treat selected diuretic resistant, volume overloaded HF patients. This approach was shown to be safe in patients with advanced HF in a small, retrospective single-center study [42], but should be tested in a large scale randomized trial. With high dose MRAs, care must be taken to avoid patients with severe renal dysfunction and serum potassium concentrations should be carefully monitored.

## 7. Use of V2-Vasopressin Receptor Blockers (Vaptans) To Treat HF

Non-osmotic secretion of arginine vasopressin due to arterial underfilling in HF patients results in hyponatremia. The demonstrataion that non-peptide vasopressin receptor antagonists cause a water diuresis and increase plasma sodium concentration in hyponatremic HF patients supports this conclusion. To the date oral tolvaptan and intravenous conivaptan are approved by the US Food and Drug Administration to treat hyponatremia in hypervolemic (HF and cirrhosis) and euvolemic (SIADH) hyponatremic patients. Conivaptan treatment in HF patients resulted in an increase in urine output and a decrease in pulmonary artery wedge pressure without affecting vascular resistance, blood pressure, heart rate or non-sodium plasma electrolytes [43]. In the several studies, tolvaptan consistently has demonstrated an aquaretic effect (urinary electrolyte-free water loss) in HF patients [44,45,46,47,48]. The EVEREST study revealed a significant improvement at one week in global clinical status and body weight reduction by adding tolvaptan to standard HF treatment in 4133 patients with decompensated systolic HF. However, long-term prognosis was not affected [49]. Less than 10% of the patients had hyponatremia. In Japan tolvaptan is approved to treat HF and the patient need not be hyponatremic. Tolvaptan can be used in association with loop diuretics.

## 8. Consequences of Diuretic Treatment

Although loop diuretics are the main agents used in treating congestion in HF patients, there are several potential negative effects attributed to their use. Overdiuresis can further diminish cardiac output in HF and, thus, reduce kidney function. Such a cardiorenal syndrome is associated with worse outcomes. Loop diuretics block sodium chloride uptake by macula densa and thereby stimulate the RAAS [50]; this could worsen the cardiac remodeling by angiotensin and aldosterone.

All diuretics, with exception of potassium-sparing ones, can cause hyperuricemia and lead to exacerbation of gout. Moreover, diuretic induced hypokalemia and hypomagnesaemia and metabolic alkalosis may predispose to cardiac arrhythmias and even sudden death. Diuretic-induced potassium wasting is a result of increased delivery of sodium and water to the aldosterone-sensitive potassium secretory site in the collecting tubules. Increased secretion of aldosterone is not uncommon due to diuretic-induced volume depletion as well as due to an underlying disease such as HF [51]. Loop diuretics inhibit the back leak of luminal potassium and therefore are kaliuretic. The generation of the positive potential in the tubular lumen with loop diuretics also increases urinary magnesium excretion [52].

Loop diuretics increase calciuresis, decrease serum calcium concentration, and may contribute to osteoporosis progression. In contrast, thiazide diuretics decrease urinary calcium excretion and thereby prevent renal calculi formation.

## 9. Diuretic Resistance

**Figure 4:**
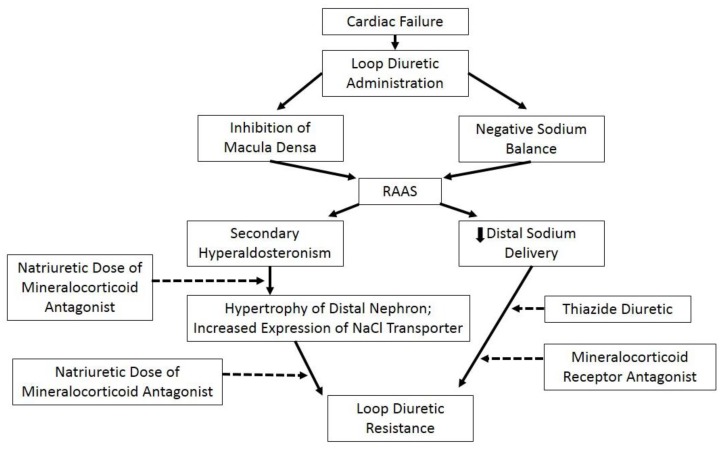
Mechanism of diuretic resistance. Reproduced with permission from [8].

Diuretic resistance in HF patients occurs when the natriuretic response to loop diuretics is impaired. Such diuretic resistance has been estimated to occur in 20% or more of patients with decompensated HF [53]. Normal subjects achieve maximum sodium excretion with 40 mg of furosemide (18). However, in HF patients the renal response to increasing doses of loop diuretics may be diminished, thus contributing to diuretic resistance. However, a widely accepted definition of loop diuretic resistance in HF patients is lacking. The characteristic features of “diuretic resistance” in patients with HF are reduced sodium delivery to the distal tubule, the site of mineralocorticoid receptors, and secondary hyperaldosteronism [54] (Figure 4).

The mechanisms of diuretic resistance are complex and differ from patient to patient. They may, however, be divided into “pharmacokinetic” and “pharmacodynamic” components [55]. The pharmacokinetic determinants of renal response to diuretics are a function of both the total amount of the drug reaching the site of action and delivery into the urine, which depends on volume of distribution, bioavailability and protein binding [56,57,58]. Response to the amount of free diuretic in tubular fluid and the amount of filtered sodium load reaching the nephron segment determine the pharmakodynamic properties and thus the effectiveness of a diuretic [59]. In HF patients with preserved renal function pharmacokinetics of loop diuretics are not altered. However, with advanced HF the arterial underfilling and concomitant neurohormonal activation increase sodium reabsorption in the proximal tubule, thus leaving a smaller amount of sodium to be blocked in the more distal nephron by diuretics that act in the distal tubule [60]. This results in reduced effectiveness of diuretics. Moreover, sodium reabsorption is also increased in the distal tubule in HF patients, an effect which contributes to diuretic resistance [61].

The fractional sodium excretion (FeNa) has been used in several studies to assess diuresis in HF patients. Baseline FeNa has been shown to be reduced to less than 1% in patients with HF and a baseline FeNa of less than 0.2% was associated with diuretic resistance [59,62].

The natriuretic response to a single dose of loop diuretic could be an early marker of loop diuretic resistance. In liver cirrhosis, a condition with a pathophysiology of water and sodium retention similar to high output HF, a natriuresis lower than 50 mEq in 8 hours after an IV 80 mg dose of furosemide was shown to be a predictor of refractory ascites [63]. Notably, elderly HF patients have been reported to have a delayed natriuretic response to diuretics. With a single dose of intravenous furosemide at a dose of 1 mg/kg, peak FeNa occurred at 30 minutes in younger patients (age17–40) and at 120 min in the patients aged 75–80 years [64]. Thus, a definition of diuretic resistance must consider the age of the patient.

Prompt recognition of diuretic resistance in patients with decompensated HF could allow for alternative strategies, such as ultrafiltration, to improve natriuresis resulting a more rapid improvement in symptoms and a shortened hospital stay.

## 10. Ultrafiltration

Ultrafiltration (UF) is another option of removing excess fluid in HF patients. As early as 1974 UF was used by Silverstein *et al.* to treat volume overloaded HF [65]. Since that time several small studies have demonstrated benefit of UF in HF patients with respect to reducing weight and dyspnea score with stable renal profile [66,67,68,69,70,71]. In 2007 the first large study on ultrafiltration in HF patients was published. The Ultrafiltration Versus Intravenous Diuretics for Patients Hospitalized for Acute Decompensated HF (UNLOAD) included 200 patients in 28 centers with decompensated volume overloaded HF. Patients in UF arm had significantly greater weight loss at 48 hours and less requirement for vasoactive drugs. Moreover, treatment with UF resulted in significantly fewer hospital readmissions due to HF during 90-day follow-up [72,73]. However, there are criticisms of this study relating to the methods used. Specifically, these included no formal protocol for diuretic use and use of a diuretic dose less than 20% of the maximum dose recommended by international guidelines for treatment of acute decompensated guidelines [3].

In one small study the response to UF was compared between HF patients with a baseline urine output of less than 1,000 mL/24 h versus those with a baseline urinary output greater than 1,000 mL/24 h. The HF patients with the lower urine output exhibited a diuresis and a fall in neurohormones with UF. These patients also had a higher right atrial pressure and lower urinary sodium excretion rate at baseline. In contrast, those HF patients with higher baseline urinary output increased their neurohormones and decreased their urine output with UF [74]. UF has been started within 24 h after admission in most small UF trials which showed benefit. Starting UF after failure of hemodynamic-guided treatment was associated with unfavorable outcome [75]. Thus, early recognition of diuretic resistance as manifested by diminished diuretic and natriuretic response to loop diuretics and a high right atrial pressure may indicate those patients most likely to have a beneficial response to UF. 

Given the problems with the UNLOAD study, a recent multicenter, controlled trial of Ultrafiltration in Decompensated HF with cardiorenal syndrome (CARRESS-HF) was undertaken in 188 patients with acute decompensated HF, increasing serum creatinine and persistent congestion. The results failed to demonstrate benefit of UF compared to a stepwise pharmacological approach [76]. Patients in both groups had the same weight loss and dyspnea score, but patients in the UF group had a significantly greater increase in serum creatinine (−0.04 *vs.* +0.23 mg/dl, *p* = 0.003) and more adverse events including bleeding and vascular complications as well as the progession of renal dysfunction (72% *vs.* 57%, *p* = 0.03). There were no differences in outcomes between the two groups during 60-days follow-up including mortality and rehospitalization. There were, however, differences in the HF patients populations in the CARRESS-HF trial compared to the UNLOAD study. The patients in the CARRESS-HF study had a higher all-cause mortality, higher baseline serum creatinine and more diabetes.

Changes in serum creatinine might not be the best end point for studies examining treatment of HF congestion with either diuretics or UF. The transient increase in serum creatinine in HF patients during fluid overload treatment may represent short-term dehydration and actually be a hall-mark of successful treatment [25,77].

UF may be indicated in some elderly HF patients with preserved left ventricular ejection fraction. These patients often have chronic kidney disease and are especially prone to repeated hospitalizations due to volume overload. Hemodynamic abnormalities in these patients may not respond as well to vasodilators or inotropes in patients with reduced ejection fraction and left ventricular dilatation. Thus, UF might be the only option for treating severe congestion in elderly patients with chronic kidney disease. Prospective randomized studies are needed to test this hypothesis. 

As with diuretics, the rate of fluid removal with UF should not exceed the rate of interstitial fluid mobilization. In patients with end-stage renal disease such fluid mobilization has been estimated to be 12–15 mL/min [78]. In patients with HF and arterial underfilling there is little information about the optimal rate of fluid mobilization, but may be lower than 12 mL/min. While UF has been proposed to remove “myocardial depressant factors” in HF patients, the small surface area of some machines in current use make removal of such cytokines inadequate [79]. Lastly, there are reports of the use of chronic peritoneal dialysis in treating refractory HF patients who are refractory to conventional therapy and have repeated rehospitalizations [80].

## 11. Conclusions

Volume overload is a hallmark for both chronic and acute decompensated HF. Because of neurohumoral activation due to arterial underfilling in HF, congestion not only causes symptoms, but may be associated with cardiac remodeling. Standard treatment with loop diuretics may not be sufficient in all cases. The addition of natriuretic doses of MRAs is a feasible option in selected HF patients. Slow continuous veno-venous ultrafiltration may also be an effective treatment of congestion for some volume overloaded HF patients, particularly in the presence of diuretic resistance. A recent excellent review about the use of UF in acute decompensated heart failure has recently been published [81].
